# High expression of *Inositol 1,4,5-trisphosphate receptor, type 2 (ITPR2)* as a novel biomarker for worse prognosis in cytogenetically normal acute myeloid leukemia

**DOI:** 10.18632/oncotarget.3024

**Published:** 2015-01-30

**Authors:** Jin-long Shi, Lin Fu, Wei-dong Wang

**Affiliations:** ^1^ Medical Engineering Support Center, Chinese PLA General Hospital, Beijing 100853, China; ^2^ Department of Hematology and Lymphoma Research Center, Peking University, Beijing 100191, China

**Keywords:** *ITPR2*, expression, prognostic biomarker, cytogenetically normal acute myeloid leukemia

## Abstract

Inositol 1,4,5-trisphosphate receptor, type 2 (*ITPR2*) is a key regulator for the activity of calcium ion transmembrane transportation, which plays a critical role in cell cycle and proliferation. However, the clinical impact of *ITPR2* in cytogenetically normal acute myeloid leukemia (CN-AML) remained unknown. Several microarray datasets were used to evaluate the association between *ITPR2* expression and clinical and molecular characteristics. *ITPR2* showed a higher expression in CN-AML patients than normal persons. In a cohort of 157 CN-AML patients, high *ITPR2* expression (*ITPR2*^high^) was associated with dramatically shorter overall survival (OS; *P* = 0.004) and event-free survival (EFS; *P* = 0.01), which were also shown in the European Leukemia Net (ELN) intermediate-I genetic category (OS: *P* = 0.0066; EFS: *P* = 0.009). Multivariable analyses adjusting for known prognostic factors confirmed *ITPR2*^high^ to be associated with shorter OS (*P* = 0.0019) and EFS (*P* = 0.012). The prognostic value of *ITPR2* was further validated in another cohort of 162 CN-AML patients (*P* = 0.007). In addition, first gene/microRNA expression signatures were derived that associated with *ITPR2*^high^ on the genome-wide scale, which provided many indications to illustrate the possible mechanisms why *ITPR2* could function. These results could aid to identify new targets and design novel therapeutic strategies for CN-AML patients.

## INTRODUCTION

Cytogenetically normal acute myeloid leukemia (CN-AML) constituting 40–50% of all AML patients is the largest cytogenetic group [1, 2]. It is characterized with the rapid accumulation of abnormal white blood cell in the bone marrow and the interface with normal blood cells' production without any chromosomal aberrations. CN-AML patients are usually categorized to be intermediate risk, yet their clinical and molecular characteristics are sharply heterogeneous. Finding proper markers has been an active area in order to obtain a more refined stratification and deeper understanding for CN-AML. Mutations of *NPM1* [3] and *CEBPA* [4] have been used as favorably prognostic biomarkers, while *FLT3-ITD* [5] and *MLL* [6] mutations have been associated with worse prognosis in the European Leukemia Net (ELN) reporting system [7]. Also, a recent report combined mutations of *DNMT3A*, *FLT3* and *NPM1* to predict clinical features for CN-AML [8]. Besides, high expression of several genes/microRNAs have been associated with adverse prognosis, including *BAALC* [9], *ERG* [9], *WT1* [10], *MN1* [11], *DNMT3B* [12], *TCF4* [13], *miR-155* [14] and *miR-3151* [15], while high expression of *LEF1* [16] has been regarded as favorable prognostic factors. Because the mechanisms of leukemogenesis are still unknown, finding new prognostic biomarkers is critical for obtaining refined risk-stratification and designing novel therapeutic strategies of CN-AML.

Activity of calcium ion transmembrane transportation is a critical biological process for maintenance and regulation of cell cycle, and plays an important role in cell proliferation, differentiation and senescence [17]. Inositol 1,4,5-trisphosphate receptor, type 2 (*ITPR2*) is a an essential regulator for mediating the mobilization of intracellular Ca2+ stores, and acts as an pivotal role in intracellular Ca2+ signaling in a variety of cell types. Early report showed that ITPR2 participated in Ca2+-calpain and Caspase-mitochondria dependent pathways and regulated the apoptosis of U937 cell [18], also *ITPR2* might act as target of *CEBPB* and all-trans retinoic acid (*ATRA*) in NB4 cells [19]. Recent reports identified *ITPR2* variations as novel susceptibility loci for renal cell carcinoma via a genome-wide association study [20]. Another report identified *ITPR2* as a susceptiable gene for Kashin-Beck disease in Han Chinese [21]. However, the prognostic impact of ITPR2 expression has not been reported in CN-AML.

This manuscript provided consolidated evidence for the first time that, high *ITPR2* expression (*ITPR2*^high^) was associated with worse prognosis in CN-AML. Firstly, *ITPR2*^high^ was shown in CN-AML patients compared to normal bone marrow (NBM) measured with microarrays. These microarray data was confirmed by qPCR [13, 16]. Secondly, the prognostic value of *ITPR2* was determined with 2 independent, relative large CN-AML cohorts, with respect to clinical, molecular characters and analysis of OS and EFS. Multivariable analysis further confirmed *ITPR2*^high^ as a worse prognostic marker. Finally, underlying mechanisms of why *ITPR2* functioned as a worse prognosticator was investigated using microarray or high-throughput sequencing data of gene/microRNA expression, genome-wide DNA methylation, combining with the known annotation and pathway information. These results may potentially facilitate our understanding of leukemogenesis and provide new criterions for risk-stratification in CN-AML, which will be finally exploited and lead to new treatment strategies.

## RESULTS

### *ITPR2* expression discriminates between CN-AML and normal bone marrow

**Figure 1:**
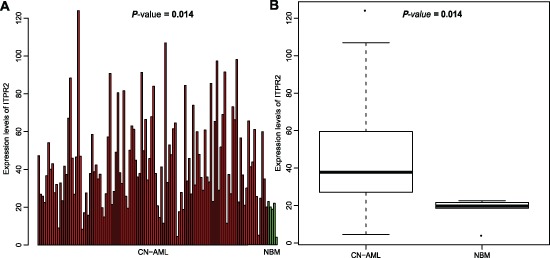
Differential expression between CN-AML and normal bone marrow **(A)** Barplot and **(B)** Boxplot of *ITPR2* expression in 116 CN-AML patients and 5 normal bone marrow samples.

Microarray analysis was used to analyze expression alteration of *ITPR2* in CN-AML (*n* = 116) and normal bone marrow (NBM) (*n* = 5). *ITPR2* was positive in these two populations. However, there existed a significant difference, *ITPR2* showed a remarkably higher expression in CN-AML than NBM (*P* = 0.014, Figure 1A and 1B). Higher expression in CN-AML made the detection of *ITPR2* more easily, and discriminated patients from normal population, these two characteristics were important for clinical applications.

### Association of *ITPR2* expression with clinical and molecular characteristics

In the 157 de novo CN-AML cohort, we found that more *ITPR2*^high^ patients fell into M1 (*P* < 0.001) and M2 (*P* = 0.018), less fell into M4 (*P* = 0.044) and M5 (*P* < 0.001) in FAB subtype, compared to *ITPR2*^low^ patients. *FLT3-ITD* mutation occurred more in *ITPR2*^high^ (*P* < 0.001) than *ITPR2*^low^ patients. In addition, *ITPR2*^high^ patients seemed more likely to have high expression of *ERG* (*P* < 0.001), *BAALC* (*P* = 0.004), *WT1* (*P* < 0.001) and *DNMT3B* (*P* < 0.001). Based on the European Leukemia Net (ELN) genetic categories, more Intermediate-I patients fell into *ITPR2*^high^ group (*P* < 0.001) (Table 1).

**Table 1:** Patients' characteristics in the primary cohort of 157 CN-AML patients according to *ITPR2* expression levels

Variable	ITPR2^high^, *n* = 78	ITPR2^low^, *n* = 79	*P*
Median age. y (range)	50.5 (18–77)	49.0 (16–73)	0.57
Female sex, no. (%)	42 (53.8)	31 (39.2)	0.079
FAB subtype, no.			
M0	1	2	1
M1	32	13	*P* < 0.001
M2	22	10	0.018
M3	1	0	0.50
M4	7	17	0.044
M5	10	29	*P* < 0.001
M6	0	1	1
Other	5	8	0.56
FLT3-ITD, no.	46	20	*P* < 0.001
FLT3-TKD, no.	7	13	0.23
NPM1, mutated no.	41	41	1
N-RAS, mutated no.	3	10	0.079
K-RAS, mutated no.	0	1	1
IDH1, mutated no.	9	10	1
IDH2, mutated no.	7	6	1
ELN genetic group, no			
Favorable	25	34	0.19
Intermediate-I	72	50	*P* < 0.001
High ERG, no.	54	24	*P* < 0.001
High BAALC, no.	48	30	0.004
High LEF1, no.	40	38	0.75
High MN1, no.	43	35	0.20
High WT1, no.	51	27	*P* < 0.001
High DNMT3B, no	55	23	*P* < 0.001
High TCF4, no	44	34	0.11

**FAB**, French-American-British classification; **ITD**, internal tandem duplication; **TKD**, tyrosine kinase domain; **ELN**, European Leukemia Net.

High *ERG*, *BAALC*, *LEF1*, *MN1*, *WT1*, *DNMT3B* and *TCF4* expression were defined as an expression level above the median of all samples, respectively.

### High expression of *ITPR2* associated with worse prognostic outcomes

**Figure 2:**
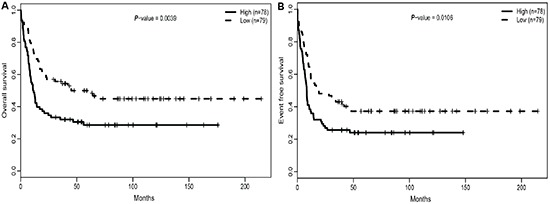
High expression of ITPR2 is associated with worse outcomes **(A)** OS and **(B)** EFS in the primary cohort of 157 CN-AML patients.

Regarding survival time as a continuous variable, *ITPR2*^high^ patients had lower median overall survival (OS: *P* = 0.0016) and event-free survival time (EFS: *P* = 0.002) (Table 2). When all 157 patients were dichotomized according to the *ITPR2* expression levels, we found that *ITPR2*^high^ group showed a significantly shorter OS (*P* = 0.0039, Figure 2A) and EFS (*P* = 0.01, Figure 2B) than *ITPR2*^low^ patient group.

**Table 2:** Survival according to *ITPR2* expression in the primary cohort of 157 CN-AML patients

Outcome	All patients, *n* = 157			ELN Favorable category			ELN Intermediate-I category		
	ITPR2^high^, *n* = 78	ITPR2^low^, *n* = 79	*P*	ITPR2^high^, *n* = 25	ITPR2^low^, *n* = 34	*P*	ITPR2^high^, *n* = 72	ITPR2^low^, *n* = 50	*P*
OS									
Median OS, m	11.28 (0.07–175.7)	39.36 (0.13–214.5)	0.0016	27.1 (0.59–163.1)	61.26 (0.3–214.5)	0.11	10.92 (0.07–175.7)	33.26 (0.13–198.7)	0.03
Estimated OS at 3 y. (95% CI)	0.32 (0.23–0.44)	0.56 (0.46–0.68)	0.03	0.48 (0.32–0.72)	0.62 (0.47–0.81)	0.26	0.31 (0.22–0.43)	0.52 (0.4–0.68)	0.04
EFS									
Median EFS, m	8.36 (0.03–148)	18.37 (0.03–214.5)	0.0022	14.36 (0.03–148)	39.59 (0.03–214.5)	0.12	8.295 (0.03–148)	15.23 (0.03–198.7)	0.02
Estimated EFS at 3 y. (95% CI)	0.26 (0.18–0.37)	0.43 (0.33–0.56)	0.05	0.4 (0.25–0.65)	0.56 (0.42–0.75)	0.25	0.24 (0.16–0.36)	0.36 (0.25–0.52)	0.05

### Prognostic value of *ITPR2* in ELN genetic subgroups

**Figure 3:**
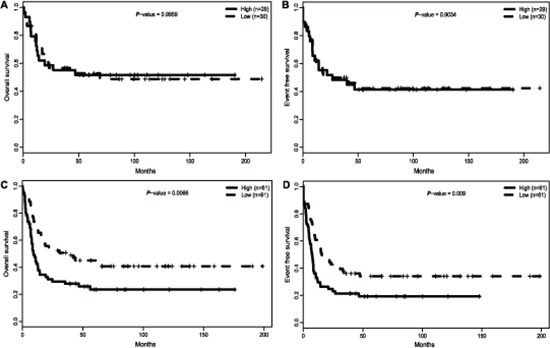
Survival of 157 CN-AML patients according to ELN genetic categories and *ITPR2* expression **(A)** OS and **(B)** EFS in the ELN favorable genetic category. **(C)** OS and **(D)** EFS in the ELN intermediate-I genetic category.

European Leukemia Net (ELN) divided CN-AML patients into the ELN favorable or ELN intermediate-I genetic categories, according to the mutation of *CEBPA*, *NPM1* and *FLT3*-*ITD* [7]. In our analysis to the primary cohort of 157 CN-AML patients, expression of *ITPR2* differed dramatically between different ELN genetic groups. More patients of the ELN Intermediate-I genetic category belonged to *ITPR2*^high^ group (*P* < 0.001, Table 1), while a trend for more patients of the ELN Favorable category belonged to *ITPR2*^low^ patient group (34 *VS* 25, *P* = 0.19, Table 1). Thus, we further investigated the impact of *ITPR2* expression within the two ELN genetic categories separately. *ITPR2* expression showed no association with OS (*P* > 0.9, Figure 3A) or EFS (*P* > 0.9, Figure 3B) in the ELN favorable category. However, in ELN intermediate-I genetic category, *ITPR2*^high^ patients showed significantly shorter OS (*P* = 0.0066, Figure 3C) and EFS (*P* = 0.009, Figure 3D) than *ITPR2*^low^ patients.

### Multivariable analysis of *ITPR2* expression associated with OS and EFS

To further determine the prognostic value of *ITPR2* expression, multivariable analysis was preformed after adjusting for the impact of other known risk factors, including commonly known mutations and several recently published prognostic factors such as *ERG* [22], *BAALC* [9], *LEF1* [16] and *WT1* [10]. In the multiple model for OS, *ITPR2*^high^ patients had 2.44 times increase of risk to death, other factors associated with longer OS included mutations of *NPM1*, *CEBPA*, and high expression of *LEF1*. In the multiple model of EFS, high expression of *ITPR2* remained a dramatically worse prognosticator (*P* = 0.012) after the adjustment of other risk factors, while mutation of *NPM1*, *CEBPA* and high expression of *LEF1* were still significantly associated with longer EFS (Table 3).

**Table 3:** Multivariable analysis with OS and EFS in the primary cohort of 157 CN-AML patients

Variable	OS, *n* = 157		EFS, *n* = 157	
	HR(95% CI)	*P*	HR(95% CI)	*P*
*ITPR2* expression, high VS low	2.44 (1.39–4.28)	0.0019	1.96 (1.16–3.31)	0.012
Age, per 10-y increase	1.11 (0.97–1.28)	0.14	1.05 (0.92–1.20)	0.49
Sex male VS female	0.80 (0.52–1.25)	0.33	0.96 (0.63–1.45)	0.83
*NPM1*, mutated VS wild type	0.50 (0.29–0.86)	0.012	0.48 (0.29–0.80)	0.0047
*CEBPA*, mutated VS wild type	0.31 (0.14–0.70)	0.0048	0.45 (0.21–0.96)	0.039
*FLT3-ITD*, mutated VS others	1.54 (0.92–2.57)	0.099	1.51 (0.92–2.48)	0.11
*IDH1*, mutated VS wild type	0.81 (0.40–1.64)	0.55	1.14 (0.60–2.14)	0.69
*IDH2*, mutated VS wild type	0.64 (0.28–1.46)	0.29	0.75 (0.32–1.74)	0.50
*EVI1*, mutated VS wild type	3.03 (0.38–24.44)	0.30	2.23 (0.28–17.70)	0.45
*ERG* expression, high VS low	1.21 (0.73–2.03)	0.46	1.21 (0.74–1.99)	0.44
*BAALC* expression, high VS low	1.14 (0.67–1.92)	0.63	0.99 (0.60–1.62)	0.96
*LEF1* expression, high VS low	0.53 (0.33–0.85)	0.0086	0.56 (0.36–0.87)	0.0098
*WT1* expression, high VS low	0.67 (0.39–1.17)	0.16	0.80 (0.48–1.35)	0.41

**HR**, hazards ratio; **CI**, confidence interval.

### Validation in an independent cohort of 162 CN-AML patients

An independent cohort of 162 de novo CN-AML patients was exploited to validate our findings. The third quartile (Q3) of *ITPR2* expression was used as the cutoff. Patients with FAB M1 were more likely to have a higher expression of *ITPR2* (*P* < 0.001), while FAB M5 seemed to be significantly associated with lower *ITPR2* expression levels (*P* = 0.0015). Further, patients with higher expression of *ITPR2* were more likely to have shorter OS (*P* = 0.011), and have higher expression of *ERG*, *WT1*, *DNMT3B* and *TCF4* (All with *P* < 0.001) and lower expression of *LEF1* (*P* = 0.004). In addition, mean OS showed significant difference between *ITPR2*^high^ and *ITPR2*^low^ groups (*P* = 0.011), and *ITPR2*^high^ patients seemed to have a shorter OS (*P* = 0.007, Figure S1.) Noticeably, mean age of *ITPR2*^low^ patients was older than that of *ITPR2*^high^ patients (*P* = 0.034), which consolidated the prognostic value of *ITPR2* expression to some extent. (All statistics were listed in Table S1.)

### Genome-wide gene-expression profiles associated with high expression of *ITPR2*

**Figure 4:**
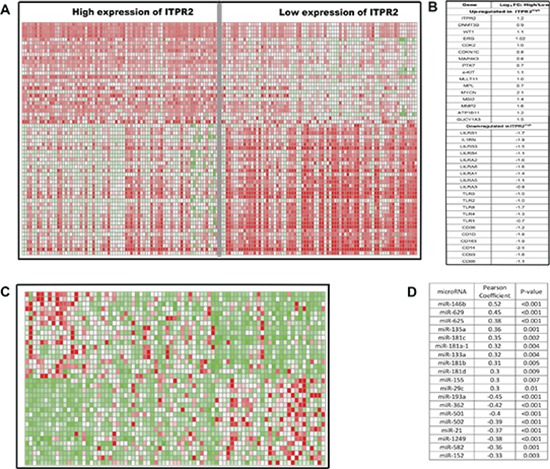
Genes/microRNAs associated with *ITPR2* expression **(A)** expression heatmap and **(B)** the list of associated genes. **(C)** expression heatmap and **(D)** the list of associated microRNAs.

To further investigate the biological role of *ITPR2*, we performed a genome-wide differential analysis based on the sample division of median *ITPR2* expression. 768 up-regulated and 1136 down-regulated genes were identified dramatically associated with high *ITPR2* expression (False Discovery Rate, FDR < 0.01; Fold Change, FC >= 1.5, data not shown). Among the 768 up-regulated genes, excitedly, we found *WT1*, *ERG* and *DNMT3B*, high expression of which were all clearly reported as worse prognostic biomarkers in CN-AML [9, 10, 12]. Other up-regulated genes included a cluster of genes that control cell cycle and differentiation (*CDK6*, *CDKN1C*, *CCND2* et.al), several genes that function as tyrosine kinase (*MAP4K3*, *PTK7* and *c-Kit*), and genes that previously known to be related with leukemogenesis (*MLLT11*, *MPL*, *MYCN*, *MSI2*). Noteworthy down-regulated genes included members of *LILR* family (*LILRB1*, *LILRB3*, *LILRB4*, *LILRA1*, *LILRA2*, *LILRA6*), members of TLR family (*TLR2*, *TLR4*, *TLR5*, *TLR7*) and the immune molecule *CD86*, which indicated the possible mechanisms of immune evasion that led to the worse outcome for CN-AML patients with high *ITPR2* expression. (See Figure 4A and 4B.)

In addition, cell signaling pathways that were associated with *ITPR2* expression alteration were identified. Based on the pathway data provided by MSigDB [23], expressions of genes that participated in a pathway were averaged to represent expression of the pathway. We found that pathways of apoptosis, antigen processing and natural killer cells mediated cytotoxicity were all significantly down-regulated (all *P* < 0.001), associated with high expression of *ITPR2*. This result, just being consistent to previously presented dysregulated genes, possibly illustrated why high expression of *ITPR2* was associated with adverse outcome in CN-AML. (See Table 4 for the statistics of those pathways.)

**Table 4:** Cell signaling pathways associated with *ITPR2* expression levels

Pathway name	According to ITPR2^high^	
	Regulation	*P*-value
KEGG_CHEMOKINE_SIGNALING_PATHWAY	Down	0.0001
KEGG_APOPTOSIS	Down	0.00018
KEGG_ANTIGEN_PROCESSING_AND_PRESENTATION	Down	< 0.0001
KEGG_NATURAL_KILLER_CELL_MEDIATED_CYTOTOXICITY	Down	< 0.0001
KEGG_FC_GAMMA_R_MEDIATED_PHAGOCYTOSIS	Down	< 0.0001

### Genome-wide microRNA profiles associated with *ITPR2* expression

To gain further insights into biological differences associated with varying *ITPR2* expression levels, genome-wide analysis of microRNA expression profiles were exploited using high throughput sequencing of 79 CN-AML patients from The Cancer Genome Atlas (*TCGA*) [24]. Sixty microRNAs were significantly associated with *ITPR2* expression (*P* < 0.01, data not shown). Of these microRNAs, *miR-155* and two members of *miR-181* family(*miR-181-a-1* and *miR-181c*) were positively associated with *ITPR2* expression, the former of which has been validated to predict worse outcome for CN-AML patients [14], and the latter were recently shown to be potential targets and associated with adverse outcomes for AML patients [25]. *MiR-193a* was the most significant microRNA negatively associated with *ITPR2* expression, which was reported to target *c-Kit* and up-regulation of *miR-193a* predicted favorable outcome in our group recently [26, 27]. (See Figure 4C and 4D.)

### Genome-wide methylation profiles associated with *ITPR2* expression

Because *ITPR2* expression was positively correlated with *DNMT3B* expression, differential analysis for methylation was performed to find different DNA methylation patterns at genome-wide scale and within main cell signaling pathways. But no clear difference was found significantly with respect to *ITPR2* expression status at the whole genome-wide level and the 186 known pathways in MSigDB [23]. (Figure S2, pathway results were not shown.)

## DISCUSSION

For the first time, we evaluated the prognostic value of *ITPR2* expression, high expression of which was associated with shorter OS and EFS in two independent, large cohorts of de novo CN-AML patients. In our study, patients with *ITPR2*^high^ are significantly more classified in the M1 or M2 FAB subgroups than with *ITPR2*^low^, suggesting that the leukemic cells of the *ITPR2*^high^ patients derive from relatively more immature cells. In addition, high *ITPR2* expression was associated with the presence of *FLT3-ITD* and high expression of *ERG*, *BAALC* and *WT1*, which were all associated with worse outcomes. Besides, more patients of high *ITPR2* expression belonged to ELN intermediate-I group (*P* < 0.001) and less to ELN-favorable group, which also showed high *ITPR2* expression as a worse biomarker. This will improve the ELN intermediate-I group risk classification and suggest that these patients may be turned candidates for alternative therapies. Furthermore, the association of high *ITPR2* expression with shorter OS and EFS was confirmed in log-rank test and multivariable analyses adjusting for almost all known molecular prognosticators in CN-AML. Considering the fact that these two cohorts of CN-AML patients received uniformed therapeutic treatments separately, these results validated *ITPR2* as an independent prognostic factor. Moreover, the fact that *ITPR2* showed higher expression in CN-AML than normal bone marrow indicated its power of discrimination and easy access, which were important characteristics for clinical application.

The mechanisms why high *ITPR2* expression is associated with adverse treatment response are unknown. However, our exploration in genome-wide gene/microRNA expression analysis provided possible interpretations. We found that expression of *WT1*, *ERG* and *DNMT3B* were significantly associated with *ITPR2*, and several genes that were active in cell cycle and tyrosine kinase process were all up-regulated, while genes that function as immune factors are down-regulated. In addition, several important cell signaling pathways showed aberrant expression associated with high expression of *ITPR2*, including the significantly down-regulation of apoptosis, natural killer cell mediated cytotoxicity and antigen processing and presentation. These changes may lead to out of control for cell death and immune escape, which might contribute to worse outcomes.

The microRNA profiles associated with *ITPR2* expression also made sense for the worse outcome. *MiR-155* was found to be positively, while *miR-193a* to be the most negatively correlated with *ITPR2* expression, because *miR-155* was clearly confirmed worse prognosticator in CN-AML and *miR-193a* was validated as a favorable biomarker by our group.

Epigenetic regulation is an important mechanism that can alter the genomic expression, however we observed no significant association between *ITPR2* expression and DNA methylation changes via our analysis of *HELP* array data. So, these CN-AML patients might show no sensitive to drugs eliminating methylation such as decitabine.

In conclusion, we show that high expression of *ITPR2* is associated with shorter OS and EFS in CN-AML patients. *ITPR2* shows higher expression in CN-AML than healthy persons, which indicates its easy access by qPCR and potential using in clinical application. However, future studies are needed to establish a standardized protocol of quantification, before it can be used for risk-stratification of CN-AML patients. Furthermore, the derived genome-wide analysis of gene/microRNA expression and DNA methylation shed light on the underlying biologic mechanisms of leukemogenesis, and might help to develop new therapeutic strategies for CN-AML disease.

## METHODS

### Patients and treatment

A cohort of 157 patients with previously untreated CN-AML (median age, 50 years, range: 16–77 years), who were collected at Erasmus University Medical Center (Rotterdam) between 1990 and 2008 [28], One hundred thirty patients (83%) were aged < 60 years (younger patients) and 27 patients (17%) were ≥ 60 years (older patients). The patients had been treated on study protocols of Dutch-Belgian Hematology-Oncology Cooperative Group (HOVON, http://www.hovon.nl). The detailed therapeutic protocol was shown in Figure S3. All samples were collected at diagnosis, with bone marrow aspirates or peripheral-blood, containing 80–100 percent blast cells after thawing [29]. Conventional cytogenetic examination of more than 20 metaphases from BM was used to determine the diagnosis of a normal karyotype. Patients were assessed for *NPM1*, *CEBPA*, *IDH1*, and *IDH2* mutations, *FLT3*-internaltandem duplications (*FLT3*-*ITD*), *N-RAS*, *K-RAS*, and *FLT3*-tyrosine kinase domain mutations (*FLT3-TKD* [D835]). This research were approved by the institutional review boards at Weill Cornell Medical College and Erasmus University Medical Center, and all subjects provided written informed consent in accordance with the Declaration of Helsinki [30]. To validate our results, another independent cohort of 162 CN-AML patients was exploited, which was provided by the multicenter AMLCG-1999 trial of the German AML Cooperative Group between 1999 and 2003 [31]. These patients received intensive double induction and consolidation chemotherapy [31]. The AMLCG-1999 clinical trials were approved by the local institutional review boards, and informed consent from all patients obtained in accordance with the Declaration of Helsinki.

### Microarray for gene expression and methylation, RNA/microRNA sequencing data

For the primary cohort of 157 CN-AML patients, pretreatment samples were studied using Affymetrix HG-U133Plus 2.0 expression GeneChips [28] and HELP methylation arrays [30], while the validating 162 patients were with Affymetrix HG-U133A expression GeneChips [31]. Experimental designs, quality control and normalization of data were carried out according to the standard Affymetrix protocols. Microarray data are available at the (*GEO*: accession no. *GSE1159*, *GSE6891* and *GSE12417* for expression, *GSE18700* for methylation) including clinical, cytogenetic and molecular characteristics [32]. To further identify microRNAs correlatively expressed with *ITPR2*, RNA-Sequencing and microRNA-Sequencing data from The Cancer Genome Atlas (*TCGA*) were exploited [24], which provided 79 CN-AML patients. Pretreatment and clinical characteristics can be publicly downloaded from *TCGA* data portal (https://tcga-data.nci.nih.gov/tcga).

### Statistical analyses

This study tried to evaluate the prognostic value of *ITPR2* expression in CN-AML, and further explored the underlying mechanisms why it functioned, based on the gene/microRNA expression and methylation data. Samples were divided into two groups, high *ITPR2* expression (*ITPR2*^high^, *n* = 78) and low *ITPR2* expression (*ITPR2*^low^, *n* = 79), based on the median expression value of *ITPR2*. Also, high and low classifications of *ERG*, *BAALC*, *WT1*, *LEF1*, *MN1*, *EVI1*, *DNMT3B* and *TCF4* were determined according to the median expression of corresponding genes. Pretreatment clinical and molecular characteristics were compared between *ITPR2*^high^ and *ITPR2*^low^ patients groups using the Fisher exact test for categories variables and the Wilcoxon rank-sum test for continuous variables. Association between *ITPR2* expression and clinical outcomes was analyzed using Kaplan-Meier method, and difference was estimated with log-rank test. Multivariable Cox proportional hazards models were used to study the time-to-event factors associated with survival endpoints.

Differential analysis was conducted with Student's *t*-test with multiple hypothesis correction (False Discovery Rate, FDR), to identify genes and pathways whose expression or methylation levels were associated with *ITPR2* expression. Pearson correlation test was performed to determine the correlated expression between *ITPR2* and microRNA sequencing profiles. All analysis was performed on the platform of R 3.1.1 software package.

## SUPPLEMENTARY FIGURES AND TABLES


