# Supplementary material for: High expression of Inositol 1,4,5-trisphosphate receptor, type 2 (ITPR2) as a novel biomarker for worse prognosis in cytogenetically normal acute myeloid leukemia
Source: Oncotarget. 2015 Jan 30;6(7):5299–309. doi: 10.18632/oncotarget.3024 (PMC4467150; doi:10.18632/oncotarget.3024)
Supplement: Supplementary file 1 [file oncotarget-06-5299-s001.pdf]

SUPPLEMENTARY FIGURES AND TABLES

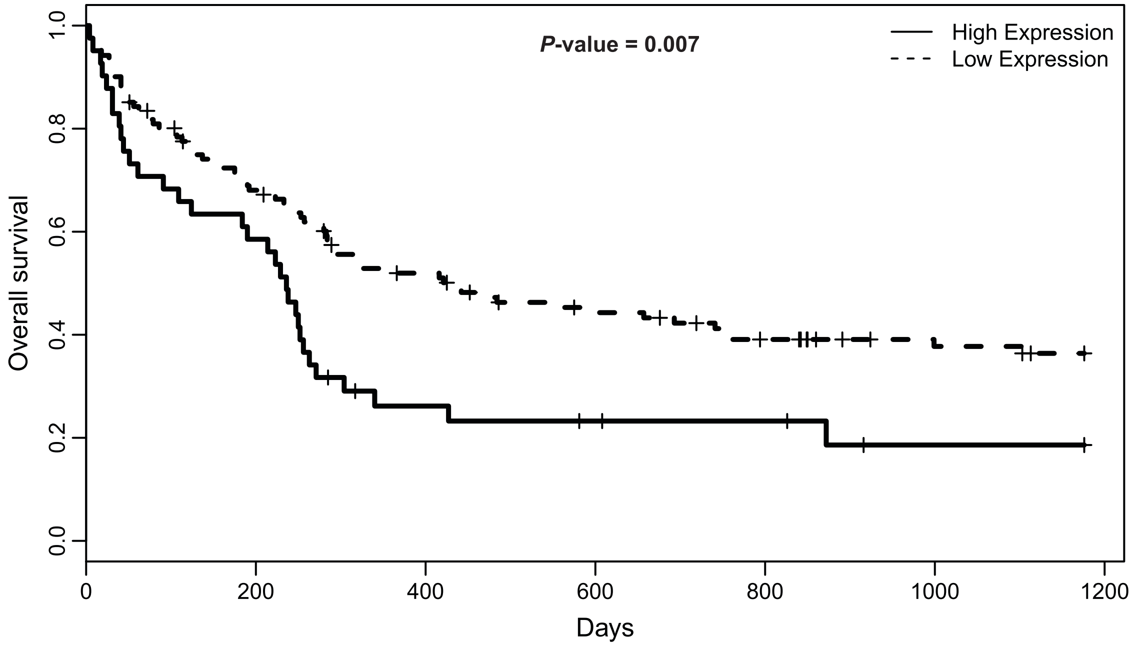

Supplementary Figure S1: High expression of *ITPR2* is associated with shorter OS in the validating cohort of 162 CN-AML patients.

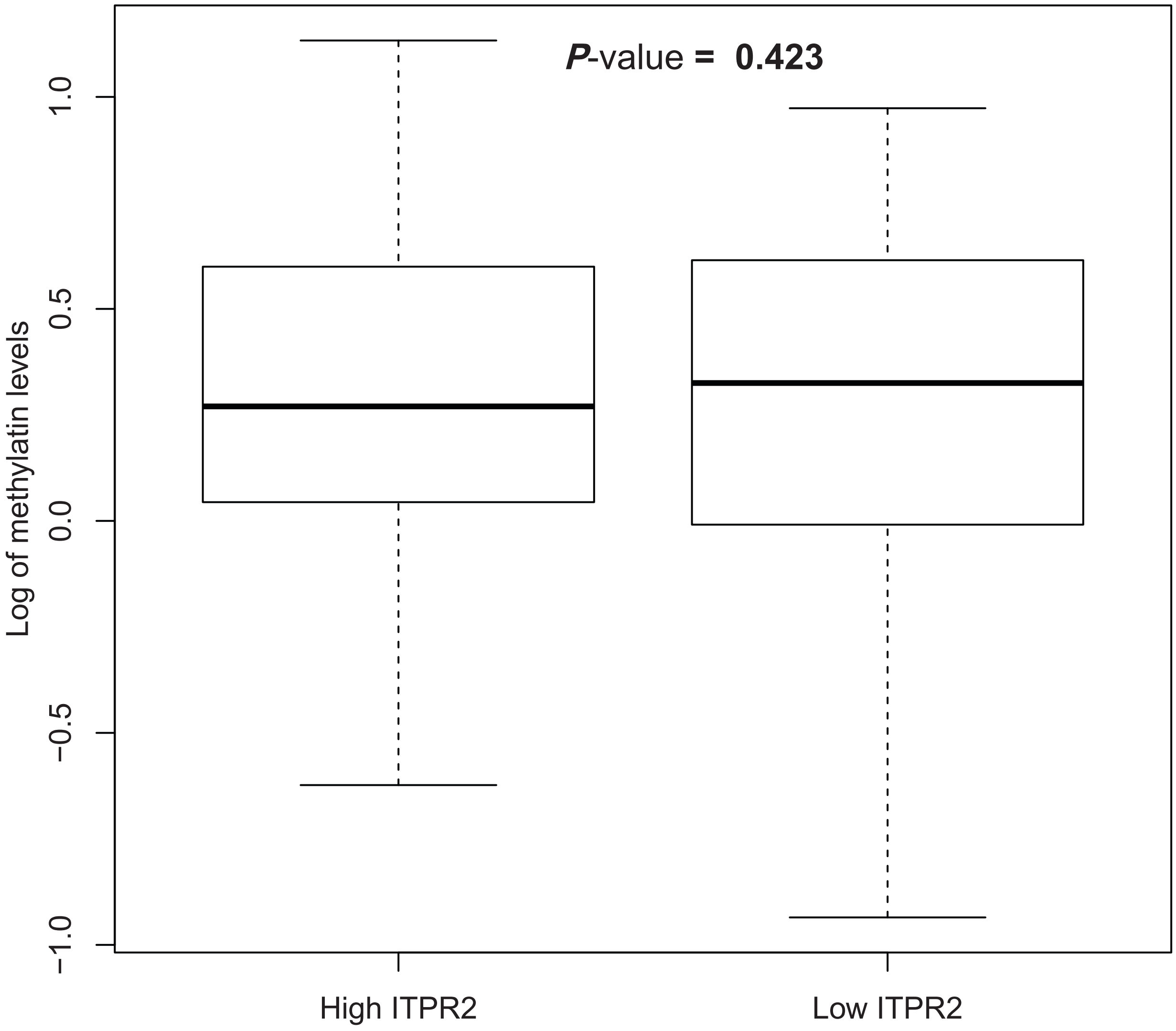

Supplementary Figure S2: Genome-wide DNA methylation in the *ITPR2*[high] and *ITPR2*[low] groups of 162 CN-AML patients.

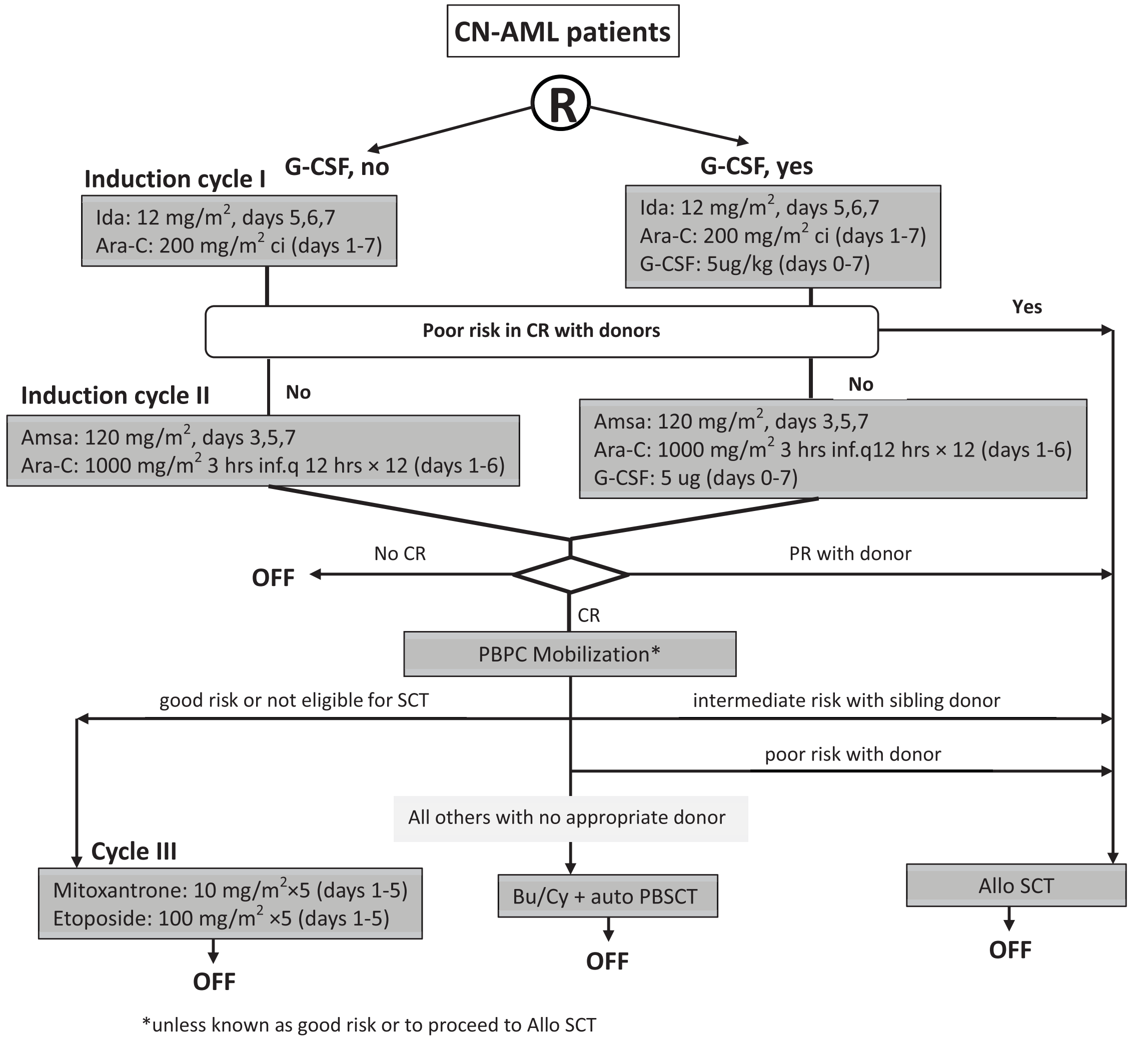

Supplementary Figure S3: Therapeutic protocols for CN-AML patients.

Supplementary Table S1: Patients' characteristics according to *ITPR2* expression levels in the validating cohort of 162 CN-AML patients

Variable	*ITPR2*^{high}, $n = 41$	*ITPR2*^{low}, $n = 121$	*P*
Median age. y (range)	53(19–75)	59(17–83)	0.034
Median OS. d (range)	236(4–1176)	314(1–1176)	0.011
FAB subtype, no (%)			
M0	0 (0)	5 (5)	0.059
M1	21 (51)	24 (20)	$P < 0.001$
M2	10 (24)	35 (29)	0.522
M3	0	0	
M4	9 (22)	33 (27)	0.511
M5	1 (2)	18 (15)	0.0015
M6	0 (0)	6 (5)	0.059
High ERG, no (%)	35 (85)	46 (38)	$P < 0.001$
High BAALC, no (%)	23 (56)	58 (48)	0.322
High LEF1, no (%)	14 (34)	67 (55)	0.004
High MN1, no (%)	22 (54)	59 (49)	0.572
High WT1, no (%)	39 (95)	42 (35)	$P < 0.001$
High DNMT3B, no (%)	35 (85)	46 (38)	$P < 0.001$
High TCF4, no (%)	29 (71)	52 (43)	$P < 0.001$

High *ERG*, *BAALC*, *LEF1*, *MN1*, *WT1*, *DNMT3B* and *TCF4* expression were defined as an expression level above the median of all samples, respectively.
